# Association of the systemic host immune response with acute hyperglycemia in mechanically ventilated septic patients

**DOI:** 10.1371/journal.pone.0248853

**Published:** 2021-03-23

**Authors:** Nauman Farooq, Byron Chuan, Hussain Mahmud, Samar R. El Khoudary, Seyed Mehdi Nouraie, John Evankovich, Libing Yang, Daniel Dunlap, William Bain, Georgios Kitsios, Yingze Zhang, Christopher P. O’Donnell, Bryan J. McVerry, Faraaz Ali Shah

**Affiliations:** 1 Division of General Internal Medicine, University of Pittsburgh, Pittsburgh, Pennsylvania, United States of America; 2 Division of Pulmonary, Allergy, and Critical Care Medicine, University of Pittsburgh, Pittsburgh, Pennsylvania, United States of America; 3 Division of Endocrinology and Metabolism, University of Pittsburgh, Pittsburgh, Pennsylvania, United States of America; 4 Department of Epidemiology, University of Pittsburgh, Pittsburgh, Pennsylvania, United States of America; 5 School of Medicine, Tsinghua University, Haidian District, Beijing, China; 6 VA Pittsburgh Healthcare System, Pittsburgh, Pennsylvania, United States of America; 7 Center for Medicine and the Microbiome, University of Pittsburgh, Pittsburgh, Pennsylvania, United States of America; Heidelberg University Hospital, GERMANY

## Abstract

Hyperglycemia during sepsis is associated with increased organ dysfunction and higher mortality. The role of the host immune response in development of hyperglycemia during sepsis remains unclear. We performed a retrospective analysis of critically ill adult septic patients requiring mechanical ventilation (n = 153) to study the relationship between hyperglycemia and ten markers of the host injury and immune response measured on the first day of ICU admission (baseline). We determined associations between each biomarker and: (1) glucose, insulin, and c-peptide levels at the time of biomarker collection by Pearson correlation; (2) average glucose and glycemic variability in the first two days of ICU admission by linear regression; and (3) occurrence of hyperglycemia (blood glucose>180mg/dL) by logistic regression. Results were adjusted for age, pre-existing diabetes mellitus, severity of illness, and total insulin and glucocorticoid dose. Baseline plasma levels of ST2 and procalcitonin were positively correlated with average blood glucose and glycemic variability in the first two days of ICU admission in unadjusted and adjusted analyses. Additionally, higher baseline ST2, IL-1ra, procalcitonin, and pentraxin-3 levels were associated with increased risk of hyperglycemia. Our results suggest associations between the host immune response and hyperglycemia in critically ill septic patients particularly implicating the interleukin-1 axis (IL-1ra), the interleukin-33 axis (ST2), and the host response to bacterial infections (procalcitonin, pentraxin-3).

## Introduction

Hyperglycemia during sepsis is associated with increased organ dysfunction and higher mortality [1–7]. Several factors contribute to hyperglycemia during sepsis including exogenous nutritional support, stress hormone release, and catecholamines and glucocorticoids administered during clinical care [8, 9], but a role for the systemic host immune response is not well defined. Preclinical studies suggest that a proinflammatory host response could increase hyperglycemia in septic patients [10–13]. Proinflammatory cytokines including interleukin-(IL-)1β, tumor necrosis factor-(TNF-) α, and IL-6 can induce insulin resistance in peripheral tissues and potentially suppress pancreatic beta cell function [14–16]. Once hyperglycemia develops, high glucose concentrations may further suppress immune cell function [17–19]. Clinical studies of the relationship between the host immune response and the occurrence of hyperglycemia in septic patients are limited and have yielded conflicting results [7, 20, 21]. Whereas some studies demonstrate a positive association between proinflammatory cytokine levels and incidence of hyperglycemia [20], others have described decreased cytokine levels in patients who develop hyperglycemia [7, 21]. Discrepancies in clinical studies may be secondary to heterogeneous patient populations, varying proportions of patients with preexisting diabetes, and differences in study duration.

We performed this study to better understand the relationship between markers of the host immune response and early glycemic control in a cohort of mechanically ventilated septic patients, a population that we postulated would undergo similar pathophysiologic changes early during critical illness. We hypothesized higher immune activation on presentation to the ICU would be associated with higher blood glucose levels in the first 2 days of ICU admission. We examined biomarkers previously associated with dysglycemia in sepsis [e.g.- interleukin (IL)-6, tumor necrosis factor receptor 1 (TNFr1), IL-1 receptor antagonist (IL-1ra)] [13, 22–25], as well as biomarkers that have been previously unexplored in this setting including markers of innate immunity [IL-8, soluble suppressor of tumorigenicity (ST)2, fractalkine], lung epithelial injury [receptor for advanced glycation end products (RAGE)], lung endothelial injury [angiopoietin-2 (Ang-2)], and the host response to bacterial infections [procalcitonin (PCT), pentraxin-3 (PTX-3)]. We determined associations between each marker and (1) measurements of glucose, insulin, c-peptide, and insulin resistance at the time of biomarker collection; (2) average glucose and glycemic variability over the first two days of intensive care unit (ICU) admission; and (3) occurrence of hyperglycemia in the first two days of ICU admission.

## Methods

### Study population

We performed a cross-sectional analysis of critically ill septic adult patients enrolled in the Acute Lung Injury Registry and Biospecimen Repository (ALIR) at the University of Pittsburgh, the details of which have been previously published [26]. Briefly, ALIR enrolls adult patients aged 18 to 90 years with acute respiratory failure requiring mechanical ventilation admitted to the medical intensive care units (ICU) at an academic tertiary care center (UPMC Presbyterian University Hospital). Exclusion criteria include inability to obtain informed consent, the presence of tracheostomy, or mechanical ventilation for more than 72 hours before the enrollment. The ALIR is approved by the University of Pittsburgh Institutional Review Board (protocol PRO10110387), and written informed consent is provided by all participants or their surrogates. All research is carried out according to The Code of Ethics of the World Medical Association (Declaration of Helsinki).

**Fig 1:**
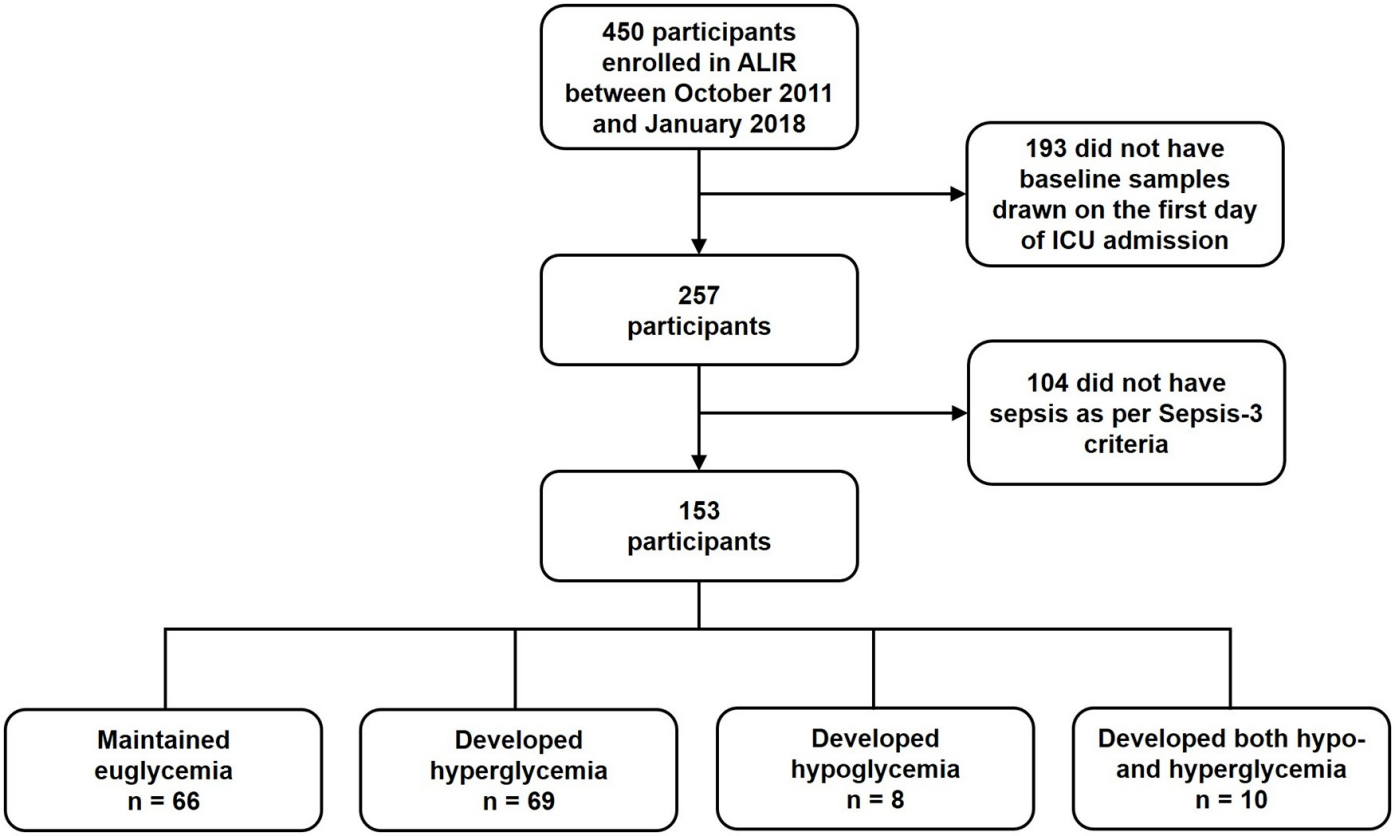
Diagram of participants included in analysis.

Baseline blood samples (defined as the first research sample collected during ICU admission) were collected from subjects within 48 hours of intubation. Baseline data for demographics, comorbidities, mechanical ventilation, physiologic and laboratory variables, prevalence of acute kidney injury at study enrollment [27], and Sequential Organ Failure Assessment (SOFA) scores (modified to exclude the neurologic component as Glasgow Coma Scales are not accurately reflective of neurological status in sedated patients and are not routinely recorded at UPMC) [28] were collected prospectively. A total of 450 medical non-trauma patients were enrolled in the ALIR between October 2011 and January 2018 (n = 450). We included patients for whom research blood samples were drawn on the first day of ICU admission (n = 257) given the dynamic nature of biomarkers and our interest in markers of early hyperglycemia. Research blood samples for participants in our study were drawn approximately 6 to 12 hours after ICU admission. We further restricted our analysis to participants who had sepsis defined by a suspected or confirmed infection with a Sequential Organ Failure Assessment (SOFA) score of at least 2, consistent with current Sepsis-3 definitions [29] (n = 153) (Fig 1).

### Markers of the systemic host injury and immune response

Baseline plasma biomarkers had been previously measured in the ALIR as part of a separate study using a customized Luminex assay (R&D Systems, Minneapolis, MN) (14). We extracted data for following biomarkers: IL-6, IL-8, TNFr1, ST2, fractalkine, RAGE, Ang-2, PCT, and PTX-3. Additional assessment of plasma IL-1ra was performed for this study using a customized Meso Scale Discovery Human U-Plex Metabolic Assay.

### Measures of glycemic control

We measured glucose, insulin, and c-peptide levels at the time of biomarker collection using banked plasma. Insulin and c-peptide were quantified using a customized Meso Scale Discovery Human U-Plex Metabolic Assay, and plasma glucose was measured using a Solo V2 glucometer (Biosense, City, ST). While glucose, insulin, and c-peptide levels measured at the time of biomarker assessment might have been influenced by exogenously administered insulin and glucose prior to study entry, few studies have characterized glycemic measures simultaneously with markers of the host immune response in septic populations. The homeostatic model of insulin resistance (HOMA-IR) was calculated by the formula: [insulin concentration (mU/L) × glucose concentration (mg/dl)]/405 [30]. Higher HOMA-IR is assumed to correlate with lower insulin sensitivity but importantly our HOMA-IR results incorporated random and not fasted values.

To assess glycemic control over the first two days of ICU admission (a time period we hypothesize would be reasonably influenced by the initial host immune response), we collected data characterizing serial blood glucose levels and the amount of insulin and glucocorticoids administered from the electronic medical record. We did not study a longer time point as we hypothesized that glycemic control after this period would be more likely to be influenced by exogenous factors. Blood glucose is monitored regularly in critically ill patients at UPMC, but choice of corrective insulin sliding scale protocols or insulin drips (if used at all) was at the discretion of treating clinicians. Glucocorticoid doses were standardized for our analyses [31].

Three measures of glycemic control were determined for each patient using all available blood glucose measurements over the first two days of ICU admission- (1) average glucose, (2) glycemic variability [estimated by the standard deviation of all available glucose values for participants with at least 3 glucose measurements (n = 127)] [32], and (3) category of glycemic control: (a) “euglycemia” [defined for this study as maintaining all blood glucose values between 70 and 179 mg/dL]; (b) “hyperglycemia” [incidence of any blood glucose greater than 180 mg/dL without an episode of hypoglycemia]; (c) “hypoglycemia” [incidence of any blood glucose less than 70 mg/dL without an episode of hyperglycemia]; and (d) “both hyperglycemia and hypoglycemia” [incidence of both hypoglycemia and hyperglycemia during the first two days]. All participants in the “both hyperglycemia and hypoglycemia” group developed hypoglycemia following insulin administration and were considered separately in our analyses.

### Statistical analysis

Statistical analyses focused on the association between baseline levels of each of the ten markers of the host response and (1) glycemic parameters at time of biomarker collection, (2) average glucose over the first two days of ICU admission, (3) glycemic variability over the first two days of ICU admission, and (4) occurrence of hyperglycemia in the first two days of ICU admission.

Data are presented as median (interquartile range) or number (%) as appropriate. Differences in baseline characteristics between the “euglycemia” and the “hyperglycemia” groups were compared by the Wilcoxon rank-sum test or Fisher’s exact test as appropriate. Average glucose, total dose of insulin administered, total dose of glucocorticoids administered, and biomarker levels were log-transformed for analysis. Association between each host response biomarker and each glycemic parameter (plasma glucose, insulin, c-peptide, and HOMA-IR) at the time of biomarker collection was compared by bivariate Pearson correlation analysis. Linear regression was utilized to assess the relationship between each biomarker and (1) average blood glucose and (2) glycemic variability over the initial 2 days of ICU admission in univariate analyses and in multivariate analyses adjusted for age, history of diabetes, SOFA score, insulin dose, and glucocorticoid dose. Logistic regression was utilized to assess the relationship between each biomarker and occurrence of hyperglycemia in univariate analyses and in multivariate analyses adjusted for age, history of diabetes, SOFA score, and glucocorticoid dose. Primary logistic regression analysis compared patients in the “hyperglycemia” to the “euglycemia” group, but sensitivity analyses were performed including patients experiencing “both hyperglycemia and hypoglycemia” in the hyperglycemia group. Finally, in exploratory analyses, relationships between biomarkers and glycemic measures (average glucose, glycemic variability, occurrence of hyperglycemia) were analyzed separately in non-diabetic and diabetic patients. The Simes method was applied to control for multiple hypothesis testing unless otherwise specified [33]. Multiplicity adjusted p values less than 0.05 were considered statistically significant. All analyses were performed in Stata 16.0 (StataCorp, College Station, TX).

## Results

### Patient characteristics

Participants in our study (n = 153) had a median age of 58.8 (IQR: 46.0–68.4) years, 67 (43.8%) were female, and most were Caucasian (92.8%), consistent with the population of patients admitted to UPMC Presbyterian. Median BMI was 29.8 (24.5–35.9) and 58 (37.1%) were known to be diabetic prior to admission (3 had type 1 diabetes mellitus). Median modified SOFA score was 7 (5–9) and 84 (54.9%) had sepsis secondary to pneumonia. Median ICU length of stay was 8 (5–12) days and 30-day mortality was 24.8%. Further characteristics are detailed in Table 1.

**Table 1:** Participant characteristics.

Variable	Entire Cohort	Euglycemic	Hyperglycemic	p-value	Hypoglycemic	Both Hypoglycemic and Hyperglycemic
**Number of participants**	153	66	69		8	10
**Demographics**						
**Age, years**	58.8 (46.0–68.4)	55.9 (35.3–65.6)	62.2 (51.9–69.9)	0.007	58.7 (50.4–66.7)	52.6 (39.9–69.1)
**Female, %**	67 (43.8)	32 (48.5)	28 (40.6)	0.358	2 (25.0)	5 (50.0)
**Caucasian, %**	142 (92.8)	63 (95.5)	67 (97.1)	0.614	5 (62.5)	7 (70.0)
**Body mass index**	29.8 (24.5–35.9)	28.1 (23.7–34.2)	32.6 (26.7–38.6)	0.006	26.5 (22.3–33.6)	25.2 (22.2–32.0)
**Comorbid conditions**						
**Diabetes mellitus, (%)**	58 (37.1)	11 (16.7)	39 (56.5)	<0.001	2 (25.0)	6 (60)
**Congestive heart failure, (%)**	15 (9.8)	7 (10.6)	8 (11.5)	0.856	0 (0)	0 (0)
**Chronic obstructive pulmonary disease, (%)**	33 (21.6)	14 (21.2)	16 (23.2)	0.783	2 (25.0)	1 (10.0)
**Chronic kidney disease, (%)**	25 (16.3)	8 (12.1)	13 (18.8)	0.283	2 (25.0)	2 (20.0)
**Chronic liver disease, (%)**	11 (7.2)	4 (6.0)	3 (4.3)	0.655	3 (37.5)	1 (10.0)
**Source of sepsis**						
**Pneumonia, (%)**	84 (54.9)	40 (60.6)	33 (47.8)	0.300	6 (75.0)	5 (50.0)
**Aspiration, (%)**	23 (15.0)	8 (12.1)	13 (18.8)		0 (0)	2 (20.0)
**Non-pulmonary, (%)**	46 (30.1)	18 (27.3)	23 (33.3)		2 (25.0)	3 (30.0)
**Severity of illness**						
**SOFA**	7 (5–9)	6.5 (4–8)	7 (5. 9)	0.184	9 (7.5–12.5)	6.5 (5–9)
**Acute kidney injury, (%)**	78 (51.0)	23 (34.9)	44 (63.8)	0.001	5 (62.5)	6 (60.0)
**Vasopressor dependent shock, (%)**	74 (48.4)	27 (40.9)	38 (55.1)	0.101	5 (62.5)	4 (40.0)
**Glycemic control during study period**						
**Average glucose, mg/dL**	141.5 (114.0–189.9)	119.1 (95.0–133.5)	192.5 (163.7–236.4)	<0.001	98.4 (89.3–108.1)	151.3 (103.6–217.4)
**Number of glucose measurements**	6 (3–11)	3 (2–6)	12 (7–16)	<0.001	9 (6–13)	17 (14–19)
**Maximum glucose, mg/dL**	183 (137–267)	136.5 (114–154)	265 (209–364)	<0.001	140.5 (127–166)	290 (199–399)
**Medications administered during study period**						
**Amount of insulin administered, IU**	0 (0–10)	0 (0–0)	10 (0–49)	<0.001	0 (0–0)	6.5 (0–40)
**Number that received glucocorticoids, (%)**	55 (35.9)	23 (34.8)	27 (39.1)	0.732	2 (25.0)	3 (30.0)
**Clinical outcomes**						
**ICU length of stay(days)**	8 (5–12)	7.5 (4–12)	8 (5–12)	0.472	6.5 (5–23)	4.5 (3–11)
**Ventilator-free days**	20 (0–24)	21 (3–25)	19 (0–24)	0.131	0 (0–23.5)	8.5 (0–24.5)
**30-day mortality (%)**	38 (24.8)	15 (19.7)	17 (24.6)	0.492	3 (37.5)	5 (50.0)

Data are presented as median (interquartile range) unless otherwise specified. p-values are for differences between euglycemia and hyperglycemia groups by Fisher’s exact test or by rank sum test as appropriate and are not adjusted for multiple comparisons. Glucocorticoid doses were standardized and are presented as doses equivalent to milligrams of prednisone. Ventilator free days were assigned as 0 for patients who died in the first 30 days of ICU admission. Abbreviations: SOFA- sequential organ failure assessment IU- international units; ICU- intensive care unit

### Glycemic control during study period

Average glucose over the first 2 days of ICU admission in the entire cohort was 141 (IQR: 114–190) mg/dL. Most patients either maintained euglycemia (66, 43%) or had hyperglycemia without hypoglycemia (69, 45%). Fewer patients had incidence of hypoglycemia alone (8, 3.2%) or experienced both hyperglycemia and hypoglycemia (10, 6.5%) (Table 1). Compared to euglycemic patients, hyperglycemic patients were older (62.2 vs 55.9 years, p = 0.007), had a higher BMI (32.6 vs 28.1 kg/m^2^, p = 0.006) and had a higher prevalence of pre-existing diabetes (56.5% vs 16.7%, p< 0.001). There was no difference in SOFA score between the two groups (p = 0.184). Hyperglycemic patients had higher mean blood glucose, a higher number of blood glucose checks, and required more insulin in the first 2 days of ICU admission compared to euglycemic patients (p<0.001 for all). Acute kidney injury (AKI) was more common in hyperglycemic patients (63.8%) as compared to euglycemic patients (34.9%, p = 0.001). There was no difference in the proportion of participants receiving glucocorticoids between the euglycemic and hyperglycemic groups (p = 0.732). Thirty-day mortality did not differ between the two groups (24.6% in hyperglycemic vs 19.7% in euglycemic group, p = 0.492).

### Host response biomarkers do not correlate with glycemic measures at the time of biomarker collection

Median plasma glucose at the time of biomarker assessment was 118 mg/dL (IQR 88–153) for the entire cohort. Plasma glucose was higher at the time of biomarker assessment in patients who experienced hyperglycemia at any point over the first 2 days of ICU admission (146 [117–218] mg/dL) compared to patients who maintained euglycemia (96 [81–124] mg/dL, p = 0.002, Table 2). Plasma insulin and HOMA-IR levels were also higher in the hyperglycemic group (p = 0.005 and p = 0.002, respectively) at the time of biomarker assessment. However, after adjusting for multiple testing, none of the host immune response biomarkers were significantly associated with glycemic measures (S1 Table).

**Table 2:** Glycemic measures at the time of biomarker collection.

Variable	Entire Cohort	Euglycemia	Hyperglycemia	p value	Hypoglycemia	Both Hyperglycemia and Hypoglycemia
**Plasma Glucose (mg/dL)**	118 (88–153)	96 (81–124)	146 (117–218)	0.002	77 (56–82)	124 (73–133)
**Plasma Insulin (μIU/mL)**	9.8 (5.4–19.7)	8.1 (5.3–13.6)	12.9 (6.8–29.6)	0.005	7.8 (5.6–17.6)	5.4 (2.2–10.7)
**Plasma C-Peptide (pg/mL)**	2375 (1270–4442)	2151 (1349–3767)	2880 (1586–6916)	0.070	1969 (1723–3912)	428 (241–928)
**HOMA-IR**	2.7 (1.4–6.5)	1.8 (1.1–3.6)	5.7 (2.2–12.4)	0.002	1.5 (1.1–2.4)	1.9 (0.4–3.4)

Data are presented as median (interquartile range). p-values are for differences between euglycemia and hyperglycemia groups by rank sum test and are adjusted for multiple comparisons. Abbreviations: IU- international units; HOMA- homeostatic model-assessment of insulin resistance.

### Increased host response biomarker levels are positively associated with average blood glucose, glycemic variability, and hyperglycemia in the first two days of ICU admission

Baseline levels of ST2 and procalcitonin (PCT) were positively associated with average blood glucose over the first 2 days of ICU admission in both unadjusted (p = 0.035 for each) and adjusted analyses (p = 0.047 and p = 0.028 respectively). None of the other ten biomarkers tested had a significant association with average blood glucose (Table 3). Positive associations between ST2 and procalcitonin and glycemic variability were also noted in unadjusted (p = 0.010 and p = 0.020 respectively) and adjusted analyses (p = 0.014 and p = 0.031, S2 Table).

**Table 3:** Unadjusted and adjusted associations of host response biomarkers with average glucose over the first two days of ICU admission.

	Unadjusted			Adjusted		
Variable	B-Coefficient	Standard Error	p-value	B-Coefficient	Standard Error	p-value
**IL-8**	-0.011	0.020	0.756			
**IL-6**	-0.015	0.014	0.437			
**TNFr1**	0.013	0.038	0.809			
**IL-1ra**	0.039	0.031	0.372			
**ST2**	0.055	0.020	0.035	0.031	0.015	0.047
**Fractalkine**	0.001	0.017	0.975			
**RAGE**	0.044	0.036	0.372			
**Ang2**	0.048	0.027	0.235			
**Procalcitonin**	0.051	0.019	0.035	0.035	0.014	0.028
**Pentraxin-3**	0.031	0.018	0.235			

Biomarker levels and average glucose were log transformed prior to analysis. Reported p-values have been adjusted for multiple comparisons. Multivariate analyses were adjusted for age, history of diabetes, total insulin dose, total glucocorticoid dose, and SOFA score. Abbreviations: ICU- intensive care unit; Ang2- angiopoietin 2; IL-6- interleukin-6; IL-8- interleukin-8; RAGE- receptor for advanced glycation end-products; ST2- suppressor of tumorigenicity 2; TNFr1- tumor-necrosis factor receptor 1.

**Fig 2:**
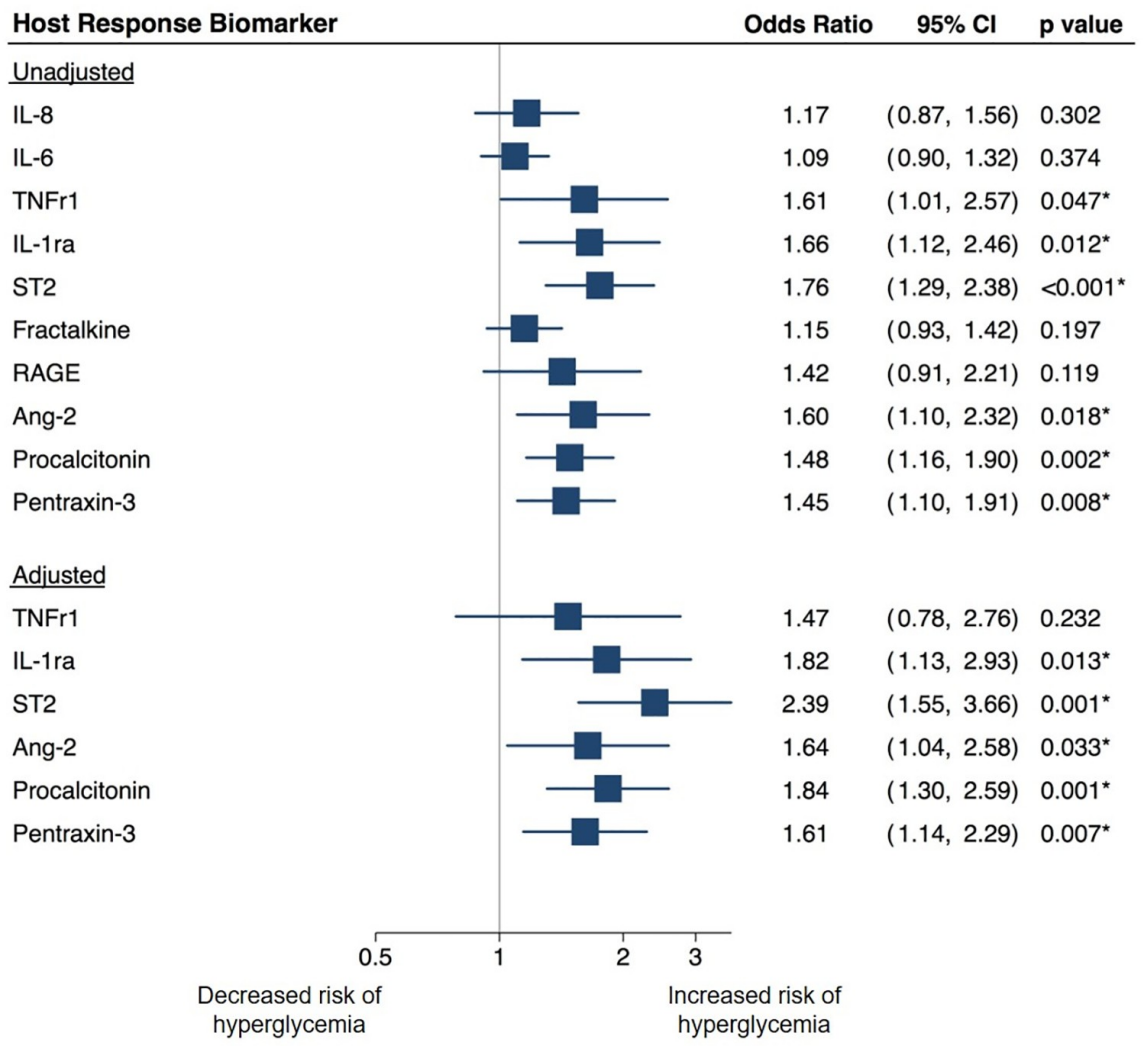
Unadjusted and adjusted associations of host response biomarkers with hyperglycemia. Participants in the “Both Hyperglycemia and Hypoglycemia” group were excluded in this analysis. Biomarker levels were log transformed prior to analysis. Reported p-values have been adjusted for multiple comparisons. Multivariate analyses were adjusted for age, history of diabetes, total glucocorticoid dose, and SOFA score. Abbreviations: Ang2: Angiopoetin 2; IL-6: Interleukin-6; IL-8: Interleukin-8; RAGE: Receptor for advanced glycation end-products; ST2: Suppressor of tumorigenicity 2; TNFr1: Tumor-necrosis factor receptor 1.

In logistic regression analyses, higher baseline levels of ST2, IL-1ra, PCT, PTX-3, and Ang-2 were associated with higher risk of hyperglycemia in the first two days of ICU admission in both unadjusted and adjusted analyses (Fig 2). In sensitivity analyses that included all patients with hyperglycemia (and did not exclude patients that experienced “both hypoglycemia and hyperglycemia”) higher ST2, IL-1ra, PCT and PTX-3 levels remained significantly associated with higher risk of hyperglycemia in both unadjusted and adjusted analyses (S1 Fig). Ang-2 was only associated with hyperglycemia in unadjusted sensitivity analyses.

Analyses of relationships between host response biomarkers and glycemic measures (average glucose, glycemic variability, and occurrence of hyperglycemia) by diabetic status suggest possible differences between non-diabetic and diabetic patients (S3–S5 Tables), but results were not robust to adjustment for multiple testing at smaller sample sizes and are exploratory at this time.

## Discussion

In this exploratory retrospective observational study of mechanically ventilated septic patients, higher host response biomarker levels early in the course of ICU admission for sepsis were associated with higher average glucose, increased glycemic variability (an independent risk factor for mortality in critically ill patients) [32], and increased risk of hyperglycemia in the first 2 days of ICU admission. Some biomarkers previously demonstrated to be associated with hyperglycemia during critical illness (IL-6, sTNFr1) were not strongly associated with average glucose or risk of hyperglycemia in our study. While statistically significant, the effect sizes observed in our study suggest that, if causally linked, the host response is attributable to only a small portion of hyperglycemia in sepsis consistent with the current multifactorial conceptual model of dysglycemia in critical illness [8].

The presence of a chronic subclinical proinflammatory state has been well-described in the setting of diabetes mellitus as activation of IL-1β, TNF-α, and IL-6 signaling pathways often precedes the onset of diabetes mellitus [34, 35]. Sepsis is marked by activation of the host immune response at a level much more acute and severe compared to diabetes mellitus [36, 37], and studies of the relationship between the host immune response and glycemic control in critically ill septic patients are both challenging and have yielded conflicting results. Studies by Leonidou et al (n = 62) and Nakamura et al (n = 153) demonstrated higher baseline IL-6 levels were associated with increased hyperglycemia in the first one and seven days of hospitalization respectively [4, 20]. In contrast, a large prospective study by van Vught et al in a cohort of almost 1000 critically ill septic patients reported that higher initial blood glucose levels were associated with lower IL-6, IL-8 and IL-10 levels in non-diabetic patients and did not correlate with cytokine levels in diabetic patients [7].

Our results do not demonstrate significant associations between baseline IL-6 and glycemic control within the first 2 days of ICU admission. Potential reasons for differences in our study include: (1) inclusion of a more restricted patient population (mechanically ventilated septic patients) compared to prior studies (all critically ill septic patients) with a higher proportion of patients with pneumonia as the inciting infection for sepsis, (2) differences in our definition of euglycemia (all observed blood glucoses between 70 and 180 mg/dL) and our study period (first 2 days of ICU admission) compared to prior studies (which ranged from an initial time point on ICU admission to the first 7 days of hospitalization), and (3) the fixed sample size which contributed to a low power for detecting significant associations when effect sizes are smaller. Notably, while our results were robust to adjustment for age, diabetic status, severity of illness, and exogenous insulin and glucocorticoid use, we acknowledge that potential imbalances in comorbid conditions or other unmeasured mechanistic pathways may confound our results.

Our results support a potential link between the IL-1 axis (IL-1ra) and glycemic control in septic patients. Interestingly, IL-1 pathway inhibitors (e.g.- anakinra) have been tested as antidiabetic agents in clinical trials [38, 39], but not in the setting of sepsis-induced hyperglycemia. Additionally, we describe novel associations between ST2 and glycemic control in critically ill septic patients. Soluble ST2, a decoy receptor for the cytokine IL-33 (a member of the IL-1 family), has previously reported roles in activating immune cells and regulating the host response although preclinical studies report conflicting results on the roles of ST2 and IL33 depending on model design and choice of septic insult [40–43]. ST2 is undetectable in normal individuals but is both elevated in septic patients and is prognostic of increased mortality [44–46]. Interestingly, elevated levels of ST2 are associated with increased risk of diabetes mellites in both non-diabetic and prediabetic patient populations [47–49], whereas IL-33 may have protective roles in glycemic control [50, 51]. To our knowledge, our study is the first to demonstrate an association between ST2 and glycemic control in sepsis.

Our study also demonstrates associations between markers of the host response to bacterial infection (PCT and PTX-3) and glycemic control in sepsis. Although typically secreted by C-cells of thyroid glands [52], PCT is also secreted by monocytes, macrophages, neuroendocrine cells, kidneys and lungs in settings of bacterial infection [53]. PTX-3 is similarly released in response to bacterial pathogens and is involved in activation of complement and other inflammatory pathways [54]. PCT and PTX-3 levels in diabetic patients have been shown to be higher when compared to non-diabetic controls [55–57], and plasma PCT is positively associated with body mass index, insulin resistance, and components of the metabolic syndrome in population-based studies [58]. In septic patients, both PCT and PTX3 have been associated with severity of sepsis, organ dysfunction, and higher mortality, but have not been studied in regard to dysglycemia [59–62]. Further studies are needed to explore potential relationships between dysglycemia and the host response to bacterial infection.

## Conclusion

In summary, our study adds additional knowledge about the pathways that may contribute not only to the pathogenesis of sepsis but also to dysglycemia, which may help inform future strategies to promote euglycemia and improve clinical outcomes in septic patients. As a single-center study specifically in a subset of septic patients requiring mechanical ventilation without external validation, our results should be interpreted as exploratory at this time.

## Supporting information

S1 Fig This sensitivity analysis includes patients in the “both hyperglycemia and hypoglycemia” group which were excluded in primary analysis. Biomarker levels were log transformed prior to analysis. Reported p-values have been adjusted for multiple comparisons. Multivariate analyses were adjusted for age, history of diabetes, total glucocorticoid dose, and SOFA score. Abbreviations: Ang2: Angiopoetin 2; IL-6: Interleukin-6; IL-8: Interleukin-8; RAGE: Receptor for advanced glycation end-products; ST2: Soluble transporter 2; TNFr1: Tumor-necrosis factor receptor 1.
(JPG)

S1 Table Correlation of host response biomarkers with glycemic parameters at the time of biomarker assessment. (DOCX)

S2 Table Unadjusted and adjusted associations of host response biomarkers with glycemic variability over the first two days of ICU admission. (DOCX)

S3 Table Associations of host response biomarkers with average glucose over the first two days of ICU admission by diabetic status. (DOCX)

S4 Table Associations of host response biomarkers with glycemic variability over the first two days of ICU admission by diabetic status. (DOCX)

S5 Table Unadjusted associations of host response biomarkers with hyperglycemia by diabetic status. (DOCX)
