# Supplementary figures and images for: Association of the systemic host immune response with acute hyperglycemia in mechanically ventilated septic patients
Source: PLoS One. 2021 Mar 23;16(3):e0248853. doi: 10.1371/journal.pone.0248853 (PMC7987165; doi:10.1371/journal.pone.0248853)

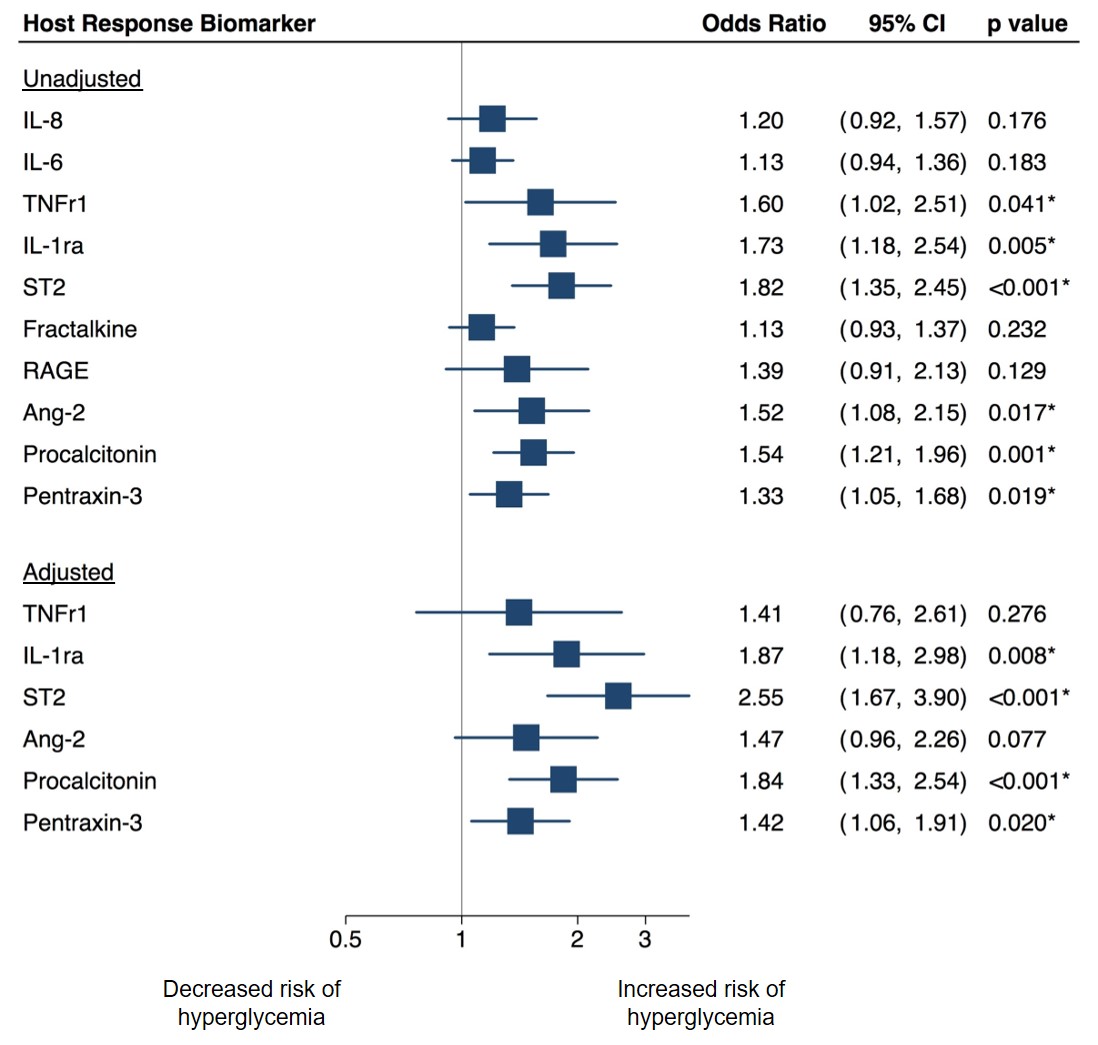

Supplement: S1 Fig — Biomarker levels were log transformed prior to analysis. Reported p-values have been adjusted for multiple comparisons. Multivariate analyses were adjusted for age, history of diabetes, total glucocorticoid dose, and SOFA score. Abbreviations: Ang2: Angiopoetin 2; IL-6: Interleukin-6; IL-8: Interleukin-8; RAGE: Receptor for advanced glycation end-products; ST2: Soluble transporter 2; TNFr1: Tumor-necrosis factor receptor 1. (JPG) [file pone.0248853.s001.jpg]
