# Supplementary material for: Association of the systemic host immune response with acute hyperglycemia in mechanically ventilated septic patients
Source: PLoS One. 2021 Mar 23;16(3):e0248853. doi: 10.1371/journal.pone.0248853 (PMC7987165; doi:10.1371/journal.pone.0248853)
Supplement: S1 Table — (DOCX) [file pone.0248853.s002.docx]

| **S1 Table. Correlation of Host Response Biomarkers with Glycemic Parameters at the Time of Biomarker Assessment.** | | | | | | | | |
| --- | --- | --- | --- | --- | --- | --- | --- | --- |
|  | **Plasma Glucose** | | **Plasma Insulin** | | **Plasma C-Peptide** | | **Plasma HOMA-IR** | |
| **Variable** | **R** | **p-value** | **r** | **p-value** | **r** | **p-value** | **r** | **p-value** |
| **Ang2** | 0.021 | 0.904 | -0.083 | 0.668 | 0.038 | 0.896 | -0.049 | 0.896 |
| **IL-8** | -0.012 | 0.939 | -0.190 | 0.413 | -0.022 | 0.904 | -0.137 | 0.582 |
| **IL-6** | -0.143 | 0.582 | -0.158 | 0.582 | -0.009 | 0.939 | -0.172 | 0.510 |
| **TNFr1** | -0.092 | 0.780 | -0.264 | 0.160 | 0.043 | 0.896 | -0.241 | 0.180 |
| **IL-1ra** | 0.025 | 0.904 | -0.040 | 0.896 | 0.133 | 0.582 | 0.001 | 0.989 |
| **ST2** | 0.056 | 0.882 | 0.034 | 0.902 | 0.086 | 0.780 | 0.072 | 0.856 |
| **Fractalkine** | -0.032 | 0.904 | -0.077 | 0.856 | 0.107 | 0.780 | -0.063 | 0.882 |
| **RAGE** | -0.083 | 0.780 | -0.125 | 0.607 | 0.046 | 0.896 | -0.122 | 0.607 |
| **Ang2** | 0.021 | 0.904 | -0.083 | 0.780 | 0.895 | 0.896 | -0.049 | 0.896 |
| **Procalcitonin** | 0.063 | 0.882 | 0.021 | 0.904 | 0.097 | 0.780 | 0.053 | 0.882 |
| **Pentraxin-3** | 0.019 | 0.911 | -0.148 | 0.582 | 0.062 | 0.882 | -0.100 | 0.780 |
| Biomarker levels and glycemic parameters were log transformed prior to analysis. Reported p values have been adjusted for multiple comparisons. Abbreviations: Ang2- angiopoietin 2; IL-1ra- interleukin-1 receptor antagonist; IL-6- interleukin-6; IL-8- interleukin-8; RAGE- receptor for advanced glycation end-products; ST2- suppressor of tumorigenicity 2; TNFr1- tumor-necrosis factor receptor 1. | | | | | | | | |
