# Supplementary material for: Association of the systemic host immune response with acute hyperglycemia in mechanically ventilated septic patients
Source: PLoS One. 2021 Mar 23;16(3):e0248853. doi: 10.1371/journal.pone.0248853 (PMC7987165; doi:10.1371/journal.pone.0248853)
Supplement: S2 Table — (DOCX) [file pone.0248853.s003.docx]

| **S2 Table. Unadjusted and Adjusted Associations of Host Response Biomarkers with Glycemic Variability over the First Two Days of ICU Admission.** | | | | | | |
| --- | --- | --- | --- | --- | --- | --- |
|  | **Unadjusted** | | | **Adjusted** | | |
| **Variable** | **B-Coefficient** | **Standard Error** | **p-value** | **B- Coefficient** | **Standard Error** | **p-value** |
| **IL-8** | 0.075 | 0.049 | 0.346 |  |  |  |
| **IL-6** | 0.018 | 0.034 | 0.603 |  |  |  |
| **TNFr1** | 0.070 | 0.899 | 0.541 |  |  |  |
| **IL-1ra** | 0.070 | 0.076 | 0.515 |  |  |  |
| **ST2** | 0.171 | 0.047 | 0.010 | 0.129 | 0.047 | 0.014 |
| **Fractalkine** | 0.029 | 0.042 | 0.541 |  |  |  |
| **RAGE** | 0.122 | 0.089 | 0.346 |  |  |  |
| **Ang-2** | 0.858 | 0.070 | 0.373 |  |  |  |
| **Procalcitonin** | 0.139 | 0.047 | 0.020 | 0.101 | 0.046 | 0.031 |
| **Pentraxin-3** | 0.061 | 0.043 | 0.346 |  |  |  |
| Biomarker levels and glycemic variability were log transformed prior to analysis. Reported p-values have been adjusted for multiple comparisons. Multivariate analyses were adjusted for age, history of diabetes, total insulin dose, total glucocorticoid dose, and SOFA score. Abbreviations: ICU- intensive care unit; Ang2- angiopoietin 2; IL-6- interleukin-6; IL-8- interleukin-8; RAGE- receptor for advanced glycation end-products; ST2- suppressor of tumorigenicity 2; TNFr1- tumor-necrosis factor receptor 1. | | | | | | |
