# Supplementary material for: Association of the systemic host immune response with acute hyperglycemia in mechanically ventilated septic patients
Source: PLoS One. 2021 Mar 23;16(3):e0248853. doi: 10.1371/journal.pone.0248853 (PMC7987165; doi:10.1371/journal.pone.0248853)
Supplement: S3 Table — (DOCX) [file pone.0248853.s004.docx]

|  | | | | | | | |
| --- | --- | --- | --- | --- | --- | --- | --- |
| **S3 Table. Associations of Host Response Biomarkers with Average Glucose over the First Two Days of ICU Admission by Diabetic Status.** | | | | | | |  |
|  | **Non-Diabetic** | | | **Diabetic** | | | |
| **Variable** | **β- Coefficient** | **Standard Error** | **p- value** | **β-Coefficient** | **Standard Error** | **p- value** | |
| **Unadjusted** |  |  |  |  |  |  | |
| **IL-8** | 0.021 | 0.019 | 0.463 | -0.055 | 0.045 | 0.458 | |
| **IL-6** | -0.001 | 0.014 | 0.946 | -0.015 | 0.030 | 0.777 | |
| **TNFr1** | -0.013 | 0.040 | 0.831 | -0.032 | 0.063 | 0.777 | |
| **IL-1ra** | 0.059 | 0.029 | 0.235 | 0.008 | 0.055 | 0.889 | |
| **ST2** | 0.059 | 0.020 | 0.040 | 0.060 | 0.034 | 0.420 | |
| **Fractalkine** | 0.019 | 0.019 | 0.474 | -0.014 | 0.028 | 0.777 | |
| **RAGE** | 0.048 | 0.035 | 0.354 | -0.017 | 0.066 | 0.885 | |
| **Ang-2** | 0.016 | 0.030 | 0.737 | 0.057 | 0.047 | 0.458 | |
| **Procalcitonin** | 0.029 | 0.019 | 0.335 | 0.090 | 0.032 | 0.060 | |
| **Pentraxin-3** | 0.039 | 0.022 | 0.266 | 0.032 | 0.026 | 0.458 | |
| Biomarker levels and glycemic variability were log transformed prior to analysis. Reported p values are adjusted for multiple comparisons. Abbreviations: ICU- intensive care unit; Ang2- angiopoietin 2; IL-1ra: interleukin-1 receptor antagonist; IL-6- interleukin-6; IL-8- interleukin-8; RAGE- receptor for advanced glycation end-products; ST2- suppressor of tumorigenicity 2; TNFr1- tumor-necrosis factor receptor 1. | | | | | | | |
