# Supplementary material for: Association of the systemic host immune response with acute hyperglycemia in mechanically ventilated septic patients
Source: PLoS One. 2021 Mar 23;16(3):e0248853. doi: 10.1371/journal.pone.0248853 (PMC7987165; doi:10.1371/journal.pone.0248853)
Supplement: S4 Table — (DOCX) [file pone.0248853.s005.docx]

|  | | | | | | | |
| --- | --- | --- | --- | --- | --- | --- | --- |
| **S4 Table.** **Associations of Host Response Biomarkers with Glycemic Variability over the First Two Days of ICU Admission by Diabetic Status.** | | | | | | |  |
|  | **Non-Diabetic** | | | **Diabetic** | | | |
| **Variable** | **β- Coefficient** | **Standard Error** | **p- value** | **β-Coefficient** | **Standard Error** | **p- value** | |
| **Unadjusted** |  |  |  |  |  |  | |
| **IL-8** | 0.118 | 0.056 | 0.078 | 0.025 | 0.099 | 0.800 | |
| **IL-6** | 0.074 | 0.040 | 0.102 | -0.035 | 0.064 | 0.935 | |
| **TNFr1** | 0.056 | 0.117 | 0.635 | 0.013 | 0.137 | 0.935 | |
| **IL-1ra** | 0.192 | 0.086 | 0.073 | -0.010 | 0.121 | 0.935 | |
| **ST2** | 0.236 | 0.058 | 0.010 | 0.169 | 0.073 | 0.130 | |
| **Fractalkine** | 0.100 | 0.054 | 0.103 | -0.010 | 0.057 | 0.935 | |
| **RAGE** | 0.229 | 0.103 | 0.073 | 0.083 | 0.146 | 0.935 | |
| **Ang-2** | 0.089 | 0.089 | 0.353 | 0.128 | 0.103 | 0.547 | |
| **Procalcitonin** | 0.129 | 0.027 | 0.073 | 0.182 | 0.071 | 0.130 | |
| **Pentraxin-3** | 0.115 | 0.066 | 0.082 | 0.080 | 0.139 | 0.463 | |
| Biomarker levels and glycemic variability were log transformed prior to analysis. Reported p values are adjusted for multiple comparisons. Abbreviations: ICU- intensive care unit; Ang2- angiopoietin 2; IL-1ra: interleukin-1 receptor antagonist; IL-6- interleukin-6; IL-8- interleukin-8; RAGE- receptor for advanced glycation end-products; ST2- suppressor of tumorigenicity 2; TNFr1- tumor-necrosis factor receptor 1. | | | | | | | |
