# Supplementary material for: Association of the systemic host immune response with acute hyperglycemia in mechanically ventilated septic patients
Source: PLoS One. 2021 Mar 23;16(3):e0248853. doi: 10.1371/journal.pone.0248853 (PMC7987165; doi:10.1371/journal.pone.0248853)
Supplement: S5 Table — (DOCX) [file pone.0248853.s006.docx]

| **S5 Table. Unadjusted Associations of Host Response Biomarkers with Hyperglycemia by Diabetic Status.** | | | | |
| --- | --- | --- | --- | --- |
|  | **Non-Diabetic** | | **Diabetic** | |
| **Variable** | **Odds Ratio (95%CI)** | **p-value** | **Odds Ratio**  **(95%CI)** | **p-value** |
| **Ang-2** | 1.47 (0.92-2.38) | 0.145 | 1.78 (0.88-3.56) | 0.265 |
| **IL-6** | 1.19 (0.94-1.51) | 0.145 | 1.12 (0.71-1.74) | 0.900 |
| **IL-8** | 1.34 (0.96-1.88) | 0.145 | 1.32 (0.58-3.03) | 0.900 |
| **Procalcitonin** | 1.61 (1.13-2.27) | 0.023 | 1.67 (1.01-2.74) | 0.170 |
| **ST2** | 2.18 (1.42-3.36) | 0.010 | 1.95 (1.00-3.82) | 0.170 |
| **Fractalkine** | 1.27 (0.93-1.72) | 0.145 | 1.07 (0.71-1.59) | 0.900 |
| **Pentraxin-3** | 1.48 (1.01-2.18) | 0.110 | 1.74 (1.05-2.89) | 0.170 |
| **RAGE** | 1.53 (0.88-2.64) | 0.129 | 1.06 (0.41-2.75) | 0.900 |
| **TNFr1** | 1.74 (0.91-3.38) | 0.145 | 1.11 (0.47-2.61) | 0.817 |
| **IL1ra** | 2.31 (1.34-4.00) | 0.015 | 1.13 (0.51-2.51) | 0.900 |
| Participants in the “Both Hyperglycemia and Hypoglycemia” group were excluded in this analysis. Host-response biomarkers were log-transformed prior to analysis. Reported p-value are adjusted for multiple comparisons. Abbreviations: Ang 2: Angiotensin 2; Fract: Fractalkine; IL-1ra: interleukin-1 receptor antagonist; IL-6: Interleukin-6; IL-8: Interleukin-8; Proc: Procalcitonin; RAGE: Receptor for advanced glycation end-products; ST2: Soluble transporter 2; TNFr1: Tumor-necrosis factor receptor 1. | | | | |
